# Infiltrating angiolipoma of the lower lip: A case report and literature review

**DOI:** 10.3892/ol.2014.2737

**Published:** 2014-11-25

**Authors:** YUICHI OHNISHI, MASAHIRO WATANABE, TOMOKO FUJII, HIROKI YASUI, HIROHITO KUBO, KENJI KAKUDO

**Affiliations:** Second Department of Oral and Maxillofacial Surgery, Osaka Dental University, Chuo-ku, Osaka 540–0008, Japan

**Keywords:** angiolipoma, lip, vascular endothelial growth factor

## Abstract

Infiltrating angiolipoma (IAL) is a rare lesion and is a clinicopathological variant of angiolipoma. IAL occurs most commonly in the trunk and extremities, it is rarely found in the head and neck regions and extremely rare in the oral cavity. This study presents the case of a 74-year-old female with IAL of the lower lip. To the best of our knowledge, this is the first case of IAL arising in the lower lip to be reported. Microscopically, IAL was unencapsulated and mature lipocytes were separated by a branching network of proliferating small vessels that infiltrated the adjacent tissues. Therefore, complete excision was difficult to perform. Magnetic resonance imaging has been reported to be valuable in determining the extent of the tumor and asserting a preoperative diagnosis. According to previous studies, the recurrence rate of IAL following surgical extirpation is 35–50%. Furthermore, the levels of mRNA expression of the vascular endothelial growth factor (VEGF) family members in the tumor were investigated. VEGF-A and -B expression were detected, however, VEGF-C and -D were expressed at extremely low levels. Excisional biopsy was performed under local anesthesia. During four years of follow-up, no evidence of tumor recurrence had been identified. An operating microscope may be utilized for the total removal of an IAL to minimize damage to normal tissues. This report indicates that mast cell-derived VEGF may be responsible for the enhanced vascularity in the tumor. We would therefore consider careful extirpation with no wide safety margin to be the procedure of choice, except when the tumor invades irregularly into the muscles.

## Introduction

Benign lipomatous tumors are classified into five types, lipoma, variants of lipoma, heterotopic lipomas, hamartomatous lesions, infiltrating or diffuse neoplastic or non-neoplastic proliferations of mature fat and hibernoma. Angiolipoma is a variant of lipoma (1). Infiltrating angiolipoma (IAL) is a rare lesion, and is a clinicopathological variant of angiolipoma, characterized by infiltration of the surrounding structures, particularly skeletal muscle. Angiolipoma accounts for 5–17% of all lipomas and predominantly presents as subcutaneous nodules in young adults, which are tender or painful on palpation, particularly during the initial growth period. Angiolipoma exists in two forms, circumscribed and diffuse. Diffuse tumors are considered to be IAL with infiltration of the surrounding muscles. IAL has a high risk of recurrence following surgical excision. Furthermore, IAL may behave in a similar manner to that of a local aggressive neoplasm. In 1966, the tumor was characterized as a clinicopathological entity by Gonzalez-Crussi *et al* (2), who reviewed the previous literature and identified cases which were consistent with this diagnosis.

According to the English literature, the tumor rarely occurs in the head and neck and is extremely rare in the oral cavity (3). Wide resection is recommended for the treatment of infiltrating angiolipomas, due to the risk of recurrence (4). The current study reports the case of a 74-year-old female with IAL of the lower lip. Following the surgical excision of the tumor, the mRNA expression levels of the vascular endothelial growth factor (VEGF) family members in the tumor were investigated. To the best of our knowledge, this is the first case of IAL arising in the lower lip to be reported. Written informed consent was obtained from the patient’s family.

## Case report

### Patient and case history

**Figure 1:**
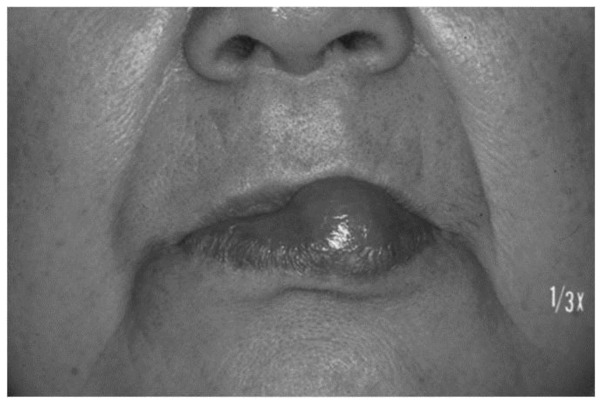
Image of patient captured at initial presentation showing a semi-firm painless mass in the lower lip.

**Figure 2:**
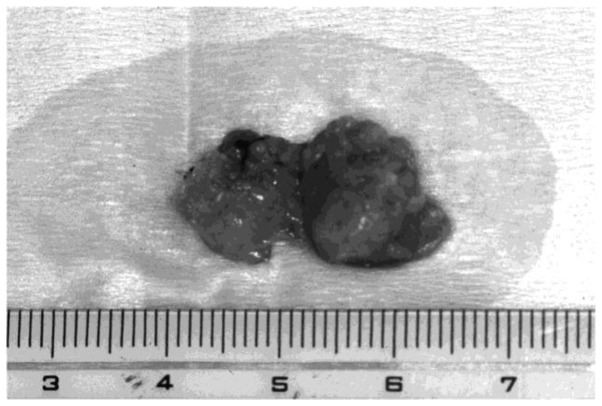
Macroscopic aspect of the cutting surface of the surgical specimen.

In April 2000, a 74-year-old female was referred to the Second Department of Oral and Maxillofacial Surgery, Osaka Dental University (Osaka, Japan) with a painless mass in the lower lip, which had been present for approximately four months. The patient had no history of facial trauma. Clinical examination revealed a relatively circumscribed soft-tissue mass of 20×19 mm in diameter in the lower lip (Fig. 1). The overlying mucosa was intact. Hematological and biochemical parameters were within the normal limits. White blood cell count, 68.6×10^2^/μl (normal range, 35.8–80.0 ×10^2^/μl); red blood cell count, 483×10^4^/μl (normal range, 380–480×10^4^/μl); hemaglobin level, 15.2 g/dl (normal range, 11.3–15.2 g/dl); hematocrit level, 44.0% (normal range, 34.0–43.0%); platelet count, 23.4×10^4^/μl (normal range, 15.0–35.0×10^4^/μl); glutamic oxaloacetic transaminase level, 22 U/l (normal range, 7–38 U/l); glutamic pyruvic transaminase level, 30 U/l (normal range, 4–44 U/l); γ-glutamyl transpeptidase, 30 U/l (normal range 9–35 U/l); creatine phosphokinase, 96 U/l (normal range, 32–187 U/l); blood urea nitrogen level, 17.5 mg/dl (normal range, 8.0–20.0 mg/dl); creatinine level, 0.51 mg/dl (normal range, 0.44–0.75 mg/dl); c-reactive protein level, 0.06 mg/dl (normal range, 0.00–0.30 mg/dl). A diagnosis of a benign tumor was determined preoperatively. Ultrasound examination was performed, however, detailed information could not be obtained due to the size of the tumor. Subsequently, an excisional biopsy was performed under local anesthesia. As the tumor was unencapsulated, the normal tissue surrounding the tumor was extirpated. The excised specimen revealed a solid soft-tissue mass (20×19×10 mm) with a dark yellow surface (Fig. 2).

**Figure 3:**
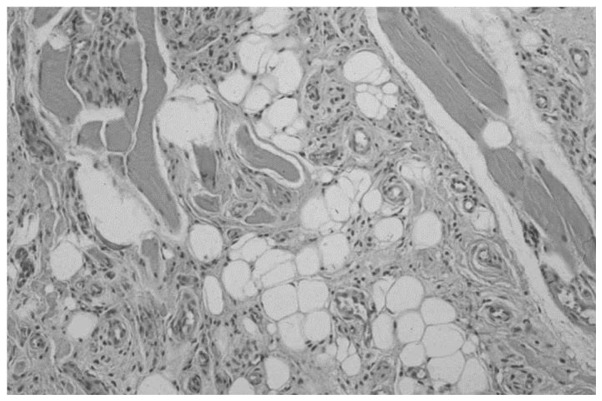
Photomicrograph shows mature lipocytes and blood vessels infiltrating cross-striated muscle fibers (hematoxylin-eosin stain, original magnification ×100).

Microscopically, the specimen was unencapsulated and mature lipocytes were separated by a branching network of proliferating small vessels that infiltrated the adjacent tissues, and muscle fibers partially existed in the tumor. It was composed of proliferating mature lipocytes and numerous small blood vessels containing microthrombi under the epithelium (Fig. 3). Cellular atypia was not observed, therefore, the pathological diagnosis of this lesion was IAL arising in the lower lip. No evidence of recurrence has been identified during four years of follow up.

**Figure 4:**
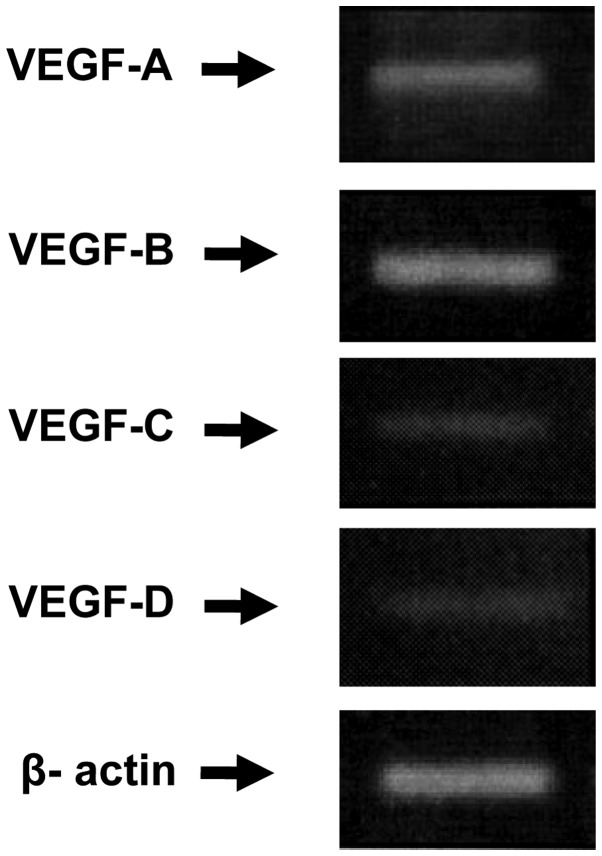
Reverse transcription-polymerase chain reaction analysis of the VEGF family members in the tumor. VEGF, vascular endothelial growth factor.

The expression of mRNA of all VEGF family members was detected in the tumor by reverse transcription (RT)-polymerase chain reaction (PCR) analysis, as shown in Fig. 4. However, the relative expression level of each of the VEGF family members differed significantly. A higher relative expression level of VEGF-A and -B were observed, when compared with VEGF-C and -D, which exhibited extremely low expression levels.

### RNA preparation and RT-PCR

Total RNA was isolated from the tissues of the patient using TRIZOL reagent (Invitrogen Life Technologies, Inc., Carlsbad, CA, USA), immediately after resection, according to the manufacturer’s instructions. A total of 10 μl RT buffer (3 mM MgCl_2_, 10 mM Tris-HCl, 75 mM KCl, 1 mM bovine serum albumin; pH 8.3) containing 1 μg RNA, 0.2 μg oligo-dT primers, 0.5 mM dNTP, 5 U of RNasin and 100 U of Moloney murine leukemia virus reverse transcriptase (Invitrogen Life Technologies, Inc.) was incubated at 37°C for 60 min and a section of each RT product was amplified by PCR, using a thermo cycler (Takara PCR Thermal Cycler Dice Gradient TP600; Takara Bio, Inc., Otsu, Japan).

The size and sequences of the primers used are shown in Table I. The PCR reactions conditions were as follows: 40 cycles of denaturation at 94°C for 1 min, annealing at 52°C for 2 min, and chain extension with Taq polymerase (Invitrogen Life Technologies, Inc.) at 72°C for 1 min, followed by a final extension step at 72°C for 20 min. Following amplification, the PCR reaction mixture was analyzed by 2% agarose gel electrophoresis and stained with ethidium bromide (Invitrogen Life Technologies, Inc.).

## Discussion

Infiltrating lipoma is a rare variant of lipoma. In 1853, Paget reported the case of a lipoma that infiltrated the trapezius muscle (2). In 1946, Regan *et al* (5) reviewed several cases and defined this entity. The tumor is most commonly identified in the deep muscles of the buttock, shoulder, thigh and extremities (6). Clinically, oral infiltrating lipoma presents as a painless solitary submucosal swelling. On palpation, the tumor is semi-firm and rubbery, with poorly defined margins. It is usually identified in the deeper tissues. Dionne and Seemayer (7) reviewed 20 patients with infiltrating lipoma and observed a recurrence rate of 62.5%.

Angiolipoma accounts for 5–17% of all lipomas and predominantly presents as subcutaneous nodules in young adults, which are tender or painful on palpation, particularly during the initial growth period (8). Microscopically, the tumor presents as yellow nodules, which consist of mature fat cells separated by a branching network of small vessels. The proportion of fatty tissue and vascular channels varies (9). The vessels commonly contain fibrin thrombi, without evidence of necrosis due to the extensive collateral circulation (8). Angiolipoma exists in two forms, circumscribed and diffuse. The circumscribed variants, with a few exceptions, are limited to the subcutis. Diffuse angiolipoma arises in the deep soft tissues and infiltrates adjacent structures and thus, complete excision is difficult (8). In the present case, the tumor was considered to be IAL and may be termed intramuscular angiolipoma, with infiltration of the surrounding muscles.

IAL occurs most commonly in the trunk and extremities (7–9), however, it is extremely rare in the oral cavity. To the best of our knowledge, only four cases of IAL of the oral cavity, including our case, have been reported in the English literature (Table II). The first and second cases of oral IAL were found in the tongue of a 49-year-old male (10) and the mucolabial fold of a 74-year-old male, respectively (11).

Lipoma is usually well encapsulated with a smooth or lobulated surface and thus, it may be resected easily, however, IAL is not encapsulated and complete excision is difficult due to the infiltration of the surrounding tissues, particularly the muscle. The recurrence rate of the tumor following surgical extirpation is 35–50% (7). The probable causes of recurrence are inaccurate preoperative estimation of the extent of the tumor and the obscure demarcation encountered during surgery (8,12). In order to clarify the extent of tumor demarcation, ultrasound provides information regarding the extent of the tumor and the infiltration of other anatomical structures. In the present case, the tumor could not be differentiated from the muscle by ultrasound. Angiography may provide more detailed information with regard to association between the vascular supply and the major vessels (13). However, magnetic resonance imaging has been reported to be more valuable than angiography or computed tomography in determining the extent of the tumor and asserting a preoperative diagnosis (12). Wide excision of the tumor has been previously reported, however, this often results in significant morbidity (14). Ida-Yonemochi *et al* (15) hypothesized that an operating microscope may be utilized for total removal of an IAL in order to minimize damage to the normal tissues during total extirpation of cerebral arteriovenous malformations (12). In the present study, the tumor was unencapsulated and infiltrated the muscle of the lower lip and thus, the tumor was extirpated carefully, including the normal tissue surrounding the tumor. A number of studies have reported the use of radiotherapy for the treatment recurrences (2,9,16). In this case, the tumor was completely excised and therefore, no further treatment was required and no recurrence was observed during the four years of postoperative follow up.

Matsuoka *et al* (12) revealed that mast cells surrounding blood vessels expressed high levels of VEGF, which is known to be an essential growth factor for endothelial cells in vasculogenesis. This result indicates that mast cell-derived VEGF may be responsible for the enhanced vascularity observed in this tumor. In the present case, VEGF-A and -B, which are known to stimulate the formation of blood vessels in tumors, were expressed. However, VEGF-C and -D, which may promote the development of lymphatic vessels in tumors and entry of tumor cells into lymphatic vessels, were expressed at extremely low levels. We hypothesize that VEGF-C and -D expression was low due to the benign nature of the tumor.

Ida-Yoncmochi *et al* (15) demonstrated that mast cells surrounding blood vessels strongly expressed VEGF, which is known to be an essential growth factor for endothelial cells in vasculogenesis. Although *in situ* hybridization was not performed in the present study, VEGF production by mast cells is highly probable as there were no other inflammatory cells within the tumor tissue. These result indicates that mast cell-derived VEGF may be responsible for the enhanced vascularity of this tumor. Therefore, we believe that IAL is associated with fat, rather than with neoplasm. We therefore recommend careful extirpation with no wide safety margin to be the procedure of choice, except in those cases where the tumor has invaded irregularly into the muscles.

In conclusion, cases of infiltrating angiolipoma of the oral cavity are extremely rare. Magnetic resonance imaging has been reported to be valuable in determining the extent of the tumor and asserting a preoperative diagnosis. Histopathology showing mature fat cells and numerous capillaries invading surrounding structure may verify the diagnosis. We consider careful extirpation with no wide safety margin to be the procedure of choice, with the exception of cases where the tumor invades irregularly into the muscles.

## Figures and Tables

**Table I:** PCR primers sequences used for reverse-transcription-PCR.

Gene	Product size, bp	Primer sequence
VEGF-A	212	5′-GCAGAATCATCACGAAGTGG-3′ 5′-GCATGGTGATGTTGGACTCC-3′
VEGF-B	246	5′-CCTTGACTGTGGAGCTCATG-3′ 5′-TGTCTGGCTTCACAGCACTG-3′
VEGF-C	435	5′-AGACTCAATGCATGCCACG-3′ 5′-TTGAGTCATCTCCAGCATCC-3′
VEGF-D	313	5′-GCTGTTGCAATGAAGAGAGC-3′ 5′-TCTTCTGTTCCAGCAAGTGG-3′
β-actin	610	5′-TGACGGGGTCACCCACACTGTGCCCATCTA-3′ 5′-CTAGAAGCATTTGCGGTGGACGATGGAGGG-3′

PCR, polymerase chain reaction; bp, base pairs; VEGF, vascular endothelial growth factor.

**Table II:** Infiltrating angiolioima of the oral cavity.

Authors (ref)	Age, years	Gender	Location	Size, cm
Lin *et al* (10)	49	Male	Tongue	3.0×2.0×2.5
Sugiura *et al* (11)	74	Male	Mucolabial fold	1.0×1.0×2.0
Dalambiris *et al* (3)	56	Female	Upper labial	1.0×1.2×0.5
Present case	74	Female	Lower lip	2.0×1.9×1.0
